# Supplementary material for: Variable and interactive effects of Sex, APOE ε4 and TREM2 on the deposition of tau in entorhinal and neocortical regions
Source: Nat Commun. 2025 Jul 1;16:5812. doi: 10.1038/s41467-025-60370-8 (PMC12214702; doi:10.1038/s41467-025-60370-8)
Supplement: Supplementary file 1 — Supplementary Information [file 41467_2025_60370_MOESM1_ESM.pdf]

Supplementary Material:

Variable and interactive effects of Sex, APOE ε4 and TREM2 on the deposition of tau in entorhinal and neocortical regions.

Joseph Giorgio, Caroline Jonson, Yilin Wang, Jennifer S. Yokoyama, Jingshen Wang, William Jagust, for the Alzheimer's Disease Neuroimaging Initiative†, The Health and Aging Brain Study (HABS-HD) Study Team[+].

†Data used in preparation of this article were obtained from the Alzheimer's Disease Neuroimaging Initiative (ADNI) database (adni.loni.usc.edu). As such, the investigators within the ADNI contributed to the design and implementation of ADNI and/or provided data but did not participate in analysis or writing of this report. A complete listing of ADNI investigators can be found in https://adni.loni.usc.edu/wpcontent/uploads/how_to_apply/ADNI_Acknowledgement_List.pdf. [+] HABS-HD MPIs: Sid E O'Bryant, Kristine Yaffe, Arthur Toga, Robert Rissman, & Leigh Johnson; and the HABS-HD Investigators: Meredith Braskie, Kevin King, James R Hall, Melissa Petersen, Raymond Palmer, Robert Barber, Yonggang Shi, Fan Zhang, Rajesh Nandy, Roderick McColl, David Mason, Bradley Christian, Nicole Philips, Stephanie Large, Joe Lee, Badri Vardarajan, Monica Rivera Mindt, Amrita Cheema, Lisa Barnes, Mark Mapstone, Annie Cohen, Amy Kind, Ozioma Okonkwo, Raul Vintimilla, Zhengyang Zhou, Michael Donohue, Rema Raman, Matthew Borzage, Michelle Mielke, Beau Ances, Ganesh Babulal, Jorge Llibre-Guerra, Carl Hill and Rocky Vig.

Supplementary Tables & Figures:

	Estimate	P-value
(Intercept)	-41.1119	0.0217
Age	0.9650	2.97e-05
SEX(female)	-1.2392	0.6860
TREM2(1)	0.0734	0.9905
APOE-ε4(1)	27.7510	< 2e-16
APOE-ε4(2)	41.9344	1.11e-09

Supplementary Table 1 Regression estimates with amyloid beta (Aβ) as the outcome in the discovery sample. Coefficient estimates and corresponding p-values from a linear regression model fitted in the discovery sample (n = 626). P-values are based on two-sided t-tests. Values in parentheses indicate the level of each genetic variable (APOE-ε4: 0, 1, or 2 alleles; TREM2: 0 = non-carrier, 1 = carrier).

	Estimate	p-value
(Intercept)	-69.1716	< 2e-16
Age	1.1613	< 2e-16
SEX(female)	5.5832	0.0040
APOE-ε4(1)	11.8967	6.46e-08
APOE-ε4(2)	24.4563	1.70e-05

Supplementary Table 2 Regression estimates with amyloid beta (Aβ) as the outcome in the replication sample. Coefficient estimates and corresponding p-values from a linear regression model fitted in the replication sample (n = 726). P-values are based on two-sided t-tests. Values in parentheses indicate the level of each genetic variable (APOE-ε4: 0, 1, or 2 alleles).

	Estimate	p-value
(Intercept)	-67.5782	5.49e-16
Age	1.1002	< 2e-16
SEX(female)	6.5551	0.0500
APOE-ε4(1)	5.1372	0.1478
APOE-ε4(2)	8.4432	0.3012
Race(Hispanic)	6.1536	0.1410
Race(White)	1.5504	0.6863
SEX(female)×Race(Hispanic)	-4.3283	0.3781
SEX(female)×Race(White)	0.6902	0.8785
APOE-ε4(1)×Race(Hispanic)	7.8882	0.1861
APOE-ε4(2)×Race(Hispanic)	47.9557	0.0015
APOE-ε4(1)×Race(White)	13.5376	0.0058
APOE-ε4(2)×Race(White)	22.0512	0.0804

Supplementary Table 3 Regression estimates with amyloid beta (Aβ) as the outcome in the replication sample including race as confound. Coefficient estimates and corresponding p-values from a linear regression model fitted in the replication sample (n = 726), including race as a covariate. P-values are based on two-sided t-tests. Values in parentheses indicate the level of each genetic variable (APOE-ε4: 0, 1, or 2 alleles).

	Estimate	p-value
(Intercept)	-34.3745	0.0619
Age	0.8937	0.0002
SEX(female)	-1.5275	0.6153
TREM2(1)	-0.9433	0.8765
APOE-ε4(1)	26.3210	9.35e-16
APOE-ε4(2)	41.7012	1.01e-09
DIAG(Dementia)	24.3724	0.0007
DIAG(MCI)	-10.8320	0.0120

Supplementary Table 4 Regression estimates with amyloid beta (Aβ) as the outcome in the discovery sample including diagnosis as confound. Coefficient estimates and corresponding p-values from a linear regression model fitted in the discovery sample (n = 623), including DIAG as a covariate. P-values are based on two-sided t-tests. Values in parentheses indicate the level of each genetic variable (APOE-ε4: 0, 1, or 2 alleles; TREM2: 0 = non-carrier, 1 = carrier).

	Estimate	p-value
(Intercept)	-70.9176	< 2e-16
Age	1.1622	< 2e-16
SEX(female)	6.2101	0.0014
APOE-ε4(1)	11.6513	9.88e-08
APOE-ε4(2)	24.0164	2.11e-05
DIAG(Dementia)	13.1098	0.0008
DIAG(MCI)	3.2123	0.1921

Supplementary Table 5 Regression estimates with amyloid beta (Aβ) as the outcome in the replication sample including diagnosis as confound. Coefficient estimates and corresponding p-values from a linear regression model fitted in the replication sample (n = 726), including DIAG as a covariate. P-values are based on two-sided t-tests. Values in parentheses indicate the level of each genetic variable (APOE-ε4: 0, 1, or 2 alleles).

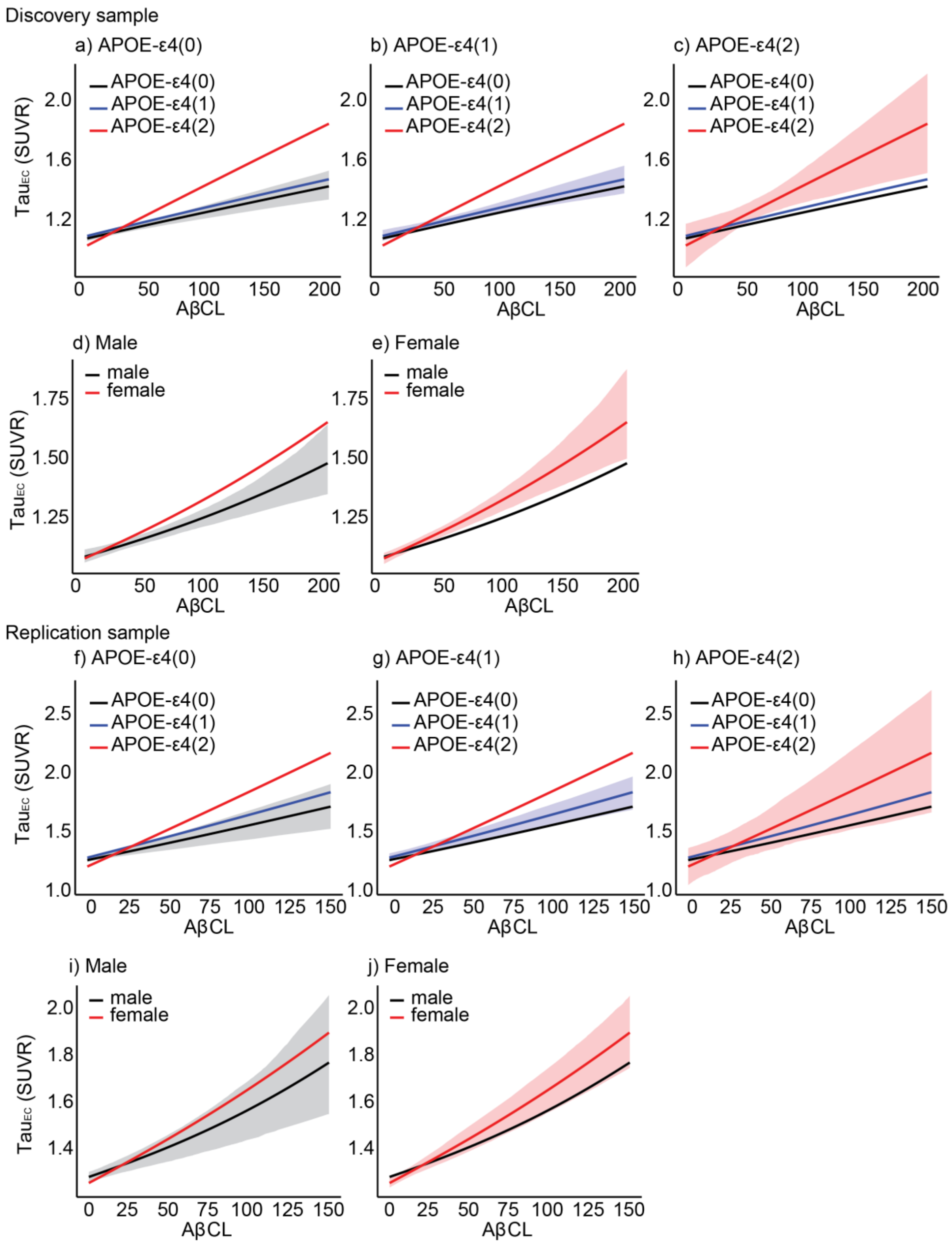

Supplementary Figure 1 Genetic influences on Entorhinal Cortex (EC) tau. Lines show the estimated marginal levels of tau pathology for individuals with varying genetic profiles at different levels of Aβ. Shaded areas represent 95% confidence intervals for the estimated trajectories, computed from 1,000 multiplier bootstrap replications (matching line color, semi-transparent). Results are shown for the discovery sample (n = 626) in panels a–e, including APOE-ε4 allele count (a–c), and Sex (d–e). Panels f–j show replication sample results (n = 726) for APOE-ε4 (f–h) and sex (i–j). Values in parentheses indicate genetic group levels (APOE-ε4: 0, 1, or 2 alleles). Source data are provided as a Source Data file.

	Estimate	P-value
(Intercept)	0.8679	< 2e-16
Aβ	0.0013	1.23e-05
Age	0.0030	0.0072
SEX(female)	-0.0071	0.7346
TREM2(1)	-0.0795	0.1017
APOE-ε4(1)	0.0283	0.2479
APOE-ε4(2)	-0.0380	0.5082
Aβ × APOE-ε4(1)	0.0001	0.7667
Aβ × APOE-ε4(2)	0.0022	0.0018
Aβ × SEX(female)	0.0008	0.0319
SEX(female) × TREM2(1)	0.1239	0.0407

Supplementary Table 6 Regression estimates with EC tau as the outcome in the discovery sample. Coefficient estimates and corresponding p-values from a linear regression model fitted in the discovery sample (n = 626). P-values are based on two-sided t-tests. Values in parentheses indicate the level of each genetic variable (APOE-ε4: 0, 1, or 2 alleles; TREM2: 0 = non-carrier, 1 = carrier).

	Estimate	p-value
(Intercept)	1.2785	< 2e-16
Aβ	0.0021	0.0002
Age	0.0001	0.8980
SEX(female)	-0.0264	0.1429
APOE-ε4(1)	0.0196	0.3585
APOE-ε4(2)	-0.0596	0.3687
Aβ×APOE-ε4(1)	0.0007	0.2421
Aβ×APOE-ε4(2)	0.0037	0.0181
Aβ×SEX(female)	0.0013	0.0321

Supplementary Table 7 Regression estimates with EC tau as the outcome in the replication sample. Coefficient estimates and corresponding p-values from a linear regression model fitted in the replication sample (n = 726). P-values are based on two-sided t-tests. Values in parentheses indicate the level of each genetic variable (APOE-ε4: 0, 1, or 2 alleles).

	Estimate	p-value
(Intercept)	1.2677	< 2e-16
Aβ	0.0020	0.0003
Age	0.0000	0.9730
SEX(female)	-0.0405	0.1597
APOE-ε4(1)	0.0258	0.4024
APOE-ε4(2)	-0.0419	0.5673
Race(Hispanic)	0.0461	0.1994
Race(White)	0.0099	0.7645
Aβ×APOE-ε4(1)	0.0006	0.3240
Aβ×APOE-ε4(2)	0.0025	0.2095
Aβ×SEX(female)	0.0013	0.0386
SEX(female)×Race(Hispanic)	-0.0011	0.9790
SEX(female)×Race(White)	0.0368	0.3485
APOE-ε4(1)×Race(Hispanic)	-0.0200	0.6967
APOE-ε4(2)×Race(Hispanic)	0.1526	0.3375
APOE-ε4(1)×Race(White)	0.0135	0.7542
APOE-ε4(2)×Race(White)	-0.0058	0.9623

Supplementary Table 8 Regression estimates with EC tau as the outcome in the replication sample including race as confound. Coefficient estimates and corresponding p-values from a linear regression model fitted in the replication sample (n = 726), including race as a covariate. P-values are based on two-sided t-tests. Values in parentheses indicate the level of each genetic variable (APOE-ε4: 0, 1, or 2 alleles).

	Estimate	p-value
(Intercept)	1.0372	< 2e-16
Aβ	0.0012	3.06e-05
Age	0.0004	0.7109
SEX(female)	-0.0008	0.9704
TREM2(1)	-0.0647	0.1631
APOE-ε4(1)	0.0309	0.1896
APOE-ε4(2)	-0.0249	0.6516
DIAG-Dementia	0.2292	8.67e-12
DIAG-MCI	0.0680	0.0006
Aβ × APOE-ε4(1)	0.0001	0.8919
Aβ × APOE-ε4(2)	0.0017	0.0116
Aβ × SEX(female)	0.0008	0.0144
SEX(female) × TREM2(1)	0.1061	0.0664

Supplementary Table 9 Regression estimates with EC tau as the outcome in the discovery sample including diagnosis as confound. Coefficient estimates and corresponding p-values from a linear regression model fitted in the discovery sample (n = 623), including DIAG as a covariate. P-values are based on two-sided t-tests. Values in parentheses indicate the level of each genetic variable (APOE-ε4: 0, 1, or 2 alleles; TREM2: 0 = non-carrier, 1 = carrier).

	Estimate	p-value
(Intercept)	1.2575	< 2e-16
Aβ	0.0020	0.0002
Age	0.0003	0.7937
SEX(female)	-0.0209	0.2494
APOE-ε4(1)	0.0207	0.3319
APOE-ε4(2)	-0.0557	0.3992
DIAG(Dementia)	0.0857	0.0102
DIAG(MCI)	0.0253	0.2247
Aβ×APOE-ε4(1)	0.0006	0.3037
Aβ×APOE-ε4(2)	0.0035	0.0236
Aβ×SEX(female)	0.0012	0.0386

Supplementary Table 10 Regression estimates with EC tau as the outcome in the replication sample including diagnosis as confound. Coefficient estimates and corresponding p-values from a linear regression model fitted in the replication sample (n = 726), including DIAG as a covariate. P-values are based on two-sided t-tests. Values in parentheses indicate the level of each genetic variable (APOE-ε4: 0, 1, or 2 alleles).

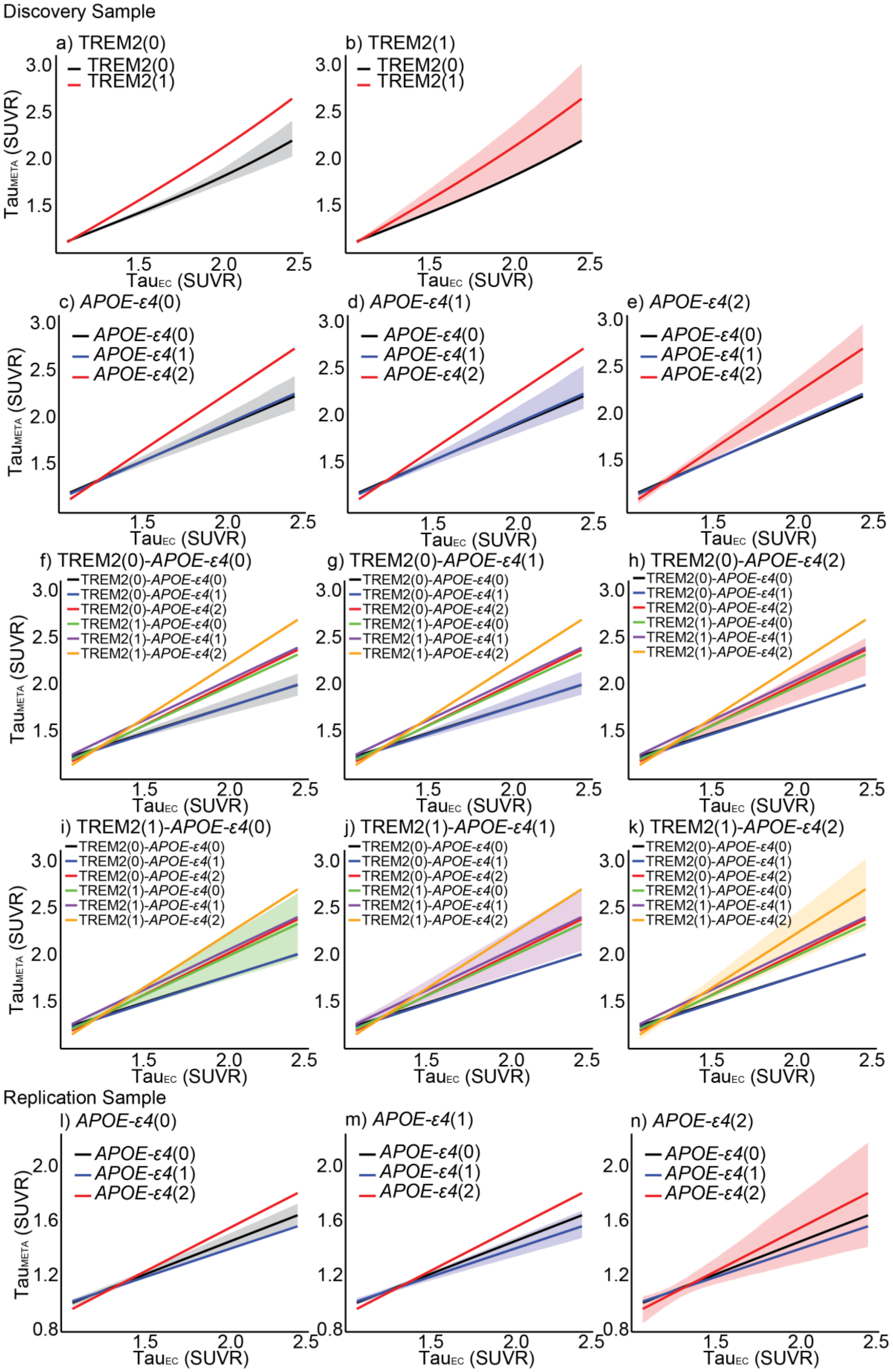

Discovery Sample
a) TREM2(0)
TREM2(0)
TREM2(1)
b) TREM2(1)
TREM2(0)
TREM2(1)
c) APOE-ε4(0)
APOE-ε4(0)
APOE-ε4(1)
APOE-ε4(2)
d) APOE-ε4(1)
APOE-ε4(0)
APOE-ε4(1)
APOE-ε4(2)
e) APOE-ε4(2)
APOE-ε4(0)
APOE-ε4(1)
APOE-ε4(2)
f) TREM2(0)-APOE-ε4(0)
g) TREM2(0)-APOE-ε4(1)
h) TREM2(0)-APOE-ε4(2)
i) TREM2(1)-APOE-ε4(0)
j) TREM2(1)-APOE-ε4(1)
k) TREM2(1)-APOE-ε4(2)
TREM2(0)-APOE-ε4(0)
TREM2(0)-APOE-ε4(1)
TREM2(0)-APOE-ε4(2)
TREM2(1)-APOE-ε4(0)
TREM2(1)-APOE-ε4(1)
TREM2(1)-APOE-ε4(2)
Replication Sample
l) APOE-ε4(0)
m) APOE-ε4(1)
n) APOE-ε4(2)
APOE-ε4(0)
APOE-ε4(1)
APOE-ε4(2)
TauMETA (SUVR)
TauEC (SUVR)
1.5
2.0
2.5
3.0
0.8
1.2
1.6
2.0

Supplementary Figure 2 Genetic influences on meta temporal tau. Lines show the estimated marginal levels of meta temporal (Tau_{Meta}) pathology for individuals with varying genetic profiles at different levels of entorhinal cortex tau (Tau_{EC}). Shaded areas represent 95% confidence intervals for the estimated trajectories, computed from 1,000 multiplier bootstrap replications (matching line color, semi-transparent). Amyloid beta (Aβ) levels were fixed at 40 centiloids to facilitate comparison across genetic groups. Results are shown for the discovery sample (n = 626) in panels a–k, including TREM2 carrier status (a–b), APOE-ε4 allele count (c–e), and interactions between TREM2 and APOE-ε4 (f–k). Panels l–n show replication results (n = 726) for APOE-ε4. Values in parentheses indicate the level of each genetic variable (APOE-ε4: 0, 1, or 2 alleles; TREM2: 0 = non-carrier, 1 = carrier). Source data are provided as a Source Data file.

	Estimate	P-value
(Intercept)	0.4039	6.19e-14
Aβ	0.0002	0.1787
EC tau	0.6809	< 2e-16
Age	-4.866e-05	0.9261
SEX(female)	0.0613	0.1407
TREM2(1)	-0.3580	0.0001
APOE-ε4(1)	-0.0406	0.3762
APOE-ε4(2)	-0.4640	8.33e-10
Aβ≥60 × EC tau	0.0194	0.0491
EC tau × APOE-ε4(1)	0.0186	0.6287
EC tau × APOE-ε4(2)	0.3643	1.65e-10
EC tau × TREM2(1)	0.3093	0.0001
TREM2(1) × APOE-ε4(1)	0.0821	0.0168
TREM2(1) × APOE4-ε4(2)	-0.0047	0.9362
Aβ × TREM2(1)	-0.0006	0.1364
EC tau × SEX(female)	-0.0504	0.1496

Supplementary Table 11 Regression estimates with MetaTemp tau as the outcome in the discovery sample. Coefficient estimates and corresponding p-values from a linear regression model fitted in the discovery sample (n = 626). Aβ≥60 × EC tau is the interaction term between an indicator variable 1{Aβ≥60} and EC tau. P-values are based on two-sided t-tests. Values in parentheses indicate the level of each genetic variable (APOE-ε4: 0, 1, or 2 alleles; TREM2: 0 = non-carrier, 1 = carrier).

	Estimate	p-value
(Intercept)	0.5491	< 2e-16
Aβ	-0.0001	0.6020
EC tau	0.4428	< 2e-16
Age	-0.0005	0.2297
SEX(female)	-0.0133	0.7411
APOE-ε4(1)	0.0816	0.0545
APOE-ε4(2)	-0.2165	0.0069
Aβ≥60×EC tau	0.1021	5.38e-14
EC tau×APOE-ε4(1)	-0.0658	0.0342
EC tau×APOE-ε4(2)	0.1567	0.0047
EC tau×SEX(female)	0.0305	0.3096

Supplementary Table 12 Regression estimates with MetaTemp tau as the outcome in the replication sample. Coefficient estimates and corresponding p-values from a linear regression model fitted in the replication sample (n = 726). Aβ≥60 × EC tau is the interaction term between an indicator variable 1{Aβ≥60} and EC tau. P-values are based on two-sided t-tests. Values in parentheses indicate the level of each genetic variable (APOE-ε4: 0, 1, or 2 alleles).

	Estimate	p-value
(Intercept)	0.5389	< 2e-16
Aβ	-0.0001	0.5381
EC tau	0.4397	< 2e-16
Age	-0.0004	0.4122
SEX(female)	-0.0121	0.7652
APOE-ε4(1)	0.0834	0.0500
APOE-ε4(2)	-0.1711	0.0372
Aβ≥60×EC tau	0.1021	7.54e-14
Race(Hispanic)	0.0122	0.4056
Race(White)	0.0021	0.8784
EC tau×APOE-ε4(1)	-0.0679	0.0311
EC tau×APOE-ε4(2)	0.1281	0.0332
EC tau×SEX(female)	0.0364	0.2296
SEX(female)×Race(Hispanic)	-0.0139	0.4227
SEX(female)×Race(White)	-0.0129	0.4161
APOE-ε4(1)×Race(Hispanic)	-0.0195	0.3511
APOE-ε4(2)×Race(Hispanic)	0.0822	0.1566
APOE-ε4(1)×Race(White)	0.0130	0.4571
APOE-ε4(2)×Race(White)	-0.0572	0.2052

Supplementary Table 13 Regression estimates with MetaTemp tau as the outcome in the replication sample including race as confound. Coefficient estimates and corresponding p-values from a linear regression model fitted in the replication sample (n = 726), including race as a covariate. Aβ≥60 × EC tau is the interaction term between an indicator variable 1{Aβ≥60}

and EC tau. P-values are based on two-sided t-tests. Values in parentheses indicate the level of each genetic variable (APOE-ε4: 0, 1, or 2 alleles).

	Estimate	p-value
(Intercept)	0.4638	< 2e-16
Aβ	0.0002	0.1324
EC tau	0.6611	< 2e-16
Age	-0.0006	0.2474
SEX(female)	0.0585	0.1608
TREM2(1)	-0.3302	0.0002
APOE-ε4(1)	-0.0210	0.6495
APOE-ε4(2)	-0.4540	1.24e-09
Aβ>60 × EC tau	0.0168	0.0865
DIAG(Dementia)	0.0671	0.0001
DIAG(MCI)	0.0116	0.2402
EC tau × APOE-ε4(1)	0.0025	0.9489
EC tau × APOE-ε4(2)	0.3545	3.30e-10
EC tau × TREM2(1)	0.2854	0.0002
TREM2(1) × APOE-ε4(1)	0.0768	0.0237
TREM2(1) × APOE-ε4(2)	-0.0055	0.9246
Aβ × TREM2(1)	-0.0006	0.1894
EC tau × SEX(female)	-0.0454	0.1958

Supplementary Table 14 Regression estimates with MetaTemp tau as the outcome in the discovery sample including diagnosis as confound. Coefficient estimates and corresponding p-values from a linear regression model fitted in the discovery sample (n = 623), including DIAG as a covariate. Aβ≥60 × EC tau is the interaction term between an indicator variable 1{Aβ≥60} and EC tau. P-values are based on two-sided t-tests. Values in parentheses indicate the level of each genetic variable (APOE-ε4: 0, 1, or 2 alleles; TREM2: 0 = non-carrier, 1 = carrier).

	Estimate	p-value
(Intercept)	0.5392	< 2e-16
Aβ	-0.0001	0.8190
EC tau	0.4405	< 2e-16
Age	-0.0004	0.3181
SEX(female)	-0.0177	0.6547
APOE-ε4(1)	0.1013	0.0158
APOE-ε4(2)	-0.1759	0.0259
Aβ≥60×EC tau	0.0929	4.28e-12
DIAG(Dementia)	0.0736	1.15e-07
DIAG(MCI)	0.0060	0.4810
EC tau×APOE-ε4(1)	-0.0809	0.0085
EC tau×APOE-ε4(2)	0.1267	0.0206
EC tau×SEX(female)	0.0360	0.2224

Supplementary Table 15 Regression estimates with MetaTemp tau as the outcome in the replication sample including diagnosis as confound. Coefficient estimates and corresponding p-values from a linear regression model fitted in the replication sample (n = 726), including DIAG as a covariate. Aβ≥60 × EC tau is the interaction term between an indicator variable 1{Aβ≥60} and EC tau. P-values are based on two-sided t-tests. Values in parentheses indicate the level of each genetic variable (APOE-ε4: 0, 1, or 2 alleles).

Part A. Weighted average mediation effects (WAME) for *APOE*-ε4 groups						
APOE-ε4	Group Size	Group Prop (100%)	WAME Size	p-value	2.5% quantile	97.5% quantile
0	353	56.3898	0.0012	0.000	0.0008	0.0016
1	239	38.1789	0.0013	0.000	0.0008	0.0018
2	34	5.4313	0.0042	0.001	0.0016	0.0070
1-0			0.0002	0.662	-0.0005	0.0008
2-0			0.0031	0.022	0.0003	0.0059
2-1			0.0029	0.033	0.0002	0.0057
Part B. Weighted average mediation effects (WAME) for *TREM2* groups						
TREM2	Group Size	Group Prop (100%)	WAME Size	p-value	2.5% quantile	97.5% quantile
0	586	93.6102	0.0013	0.000	0.0010	0.0016
1	40	6.3898	0.0021	0.000	0.0014	0.0028
1-0			0.0008	0.014	0.0001	0.0013
Part C. Weighted average mediation effects (WAME) for SEX groups						
SEX	Group Size	Group Prop (100%)	WAME Size	p-value	2.5% quantile	97.5% quantile
Male	290	46.3259	0.0011	0.000	0.0007	0.0016
Female	336	53.6741	0.0016	0.000	0.0012	0.0020
Female-Male			0.0005	0.110	-0.0002	0.0011

Supplementary Table 16 Mediation effects of APOE-ε4, TREM2, and sex in the discovery sample (n = 626). Mediation effects for the $A\beta \rightarrow Tau_{EC} \rightarrow Tau_{Meta}$ pathway are shown across groups defined by APOE-ε4 allele count (0,1,2) (Part A), TREM2 risk variant carrier status (0 = non-carrier, 1 = carrier) (Part B), and sex (Part C). Weighted average mediation effects (WAME) were computed for each group by aggregating fully stratified estimates using empirical sample proportions. Values in parentheses indicate the level of each genetic variable (APOE-ε4: 0, 1, or 2 alleles; TREM2: 0 = non-carrier, 1 = carrier). Pairwise contrasts (e.g., 1–0, 2–0, 2–1) indicate between-group differences; for instance, APOE-ε4 = 1–0 in Part A represents the WAME for APOE-ε4 heterozygotes minus that for non-carriers. Two-sided p-values and two-sided 95% confidence intervals were computed using 1,000 multiplier bootstrap replications.

Part A. Weighted average mediation effects (WAME) for *APOE*-ε4 groups

APOE-ε4	Group Size	Group Prop (100%)	WAME Size	one-sided p-value	5% quantile	100% quantile
0	523	72.04	0.0013	0.001	0.0008	0.0027
1	182	25.07	0.0015	0.000	0.0010	0.0024
2	21	2.89	0.0040	0.058	0.0011	0.0128
1-0			0.0001	0.389	-0.0006	0.0017
2-0			0.0026	0.135	-0.0004	0.0116
2-1			0.0025	0.151	-0.0005	0.0112

Part B. Weighted average mediation effects (WAME) for SEX groups

SEX	Group Size	Group Prop (100%)	WAME Size	one-sided p-value	5% quantile	100% quantile
Male	293	40.36	0.0011	0.001	0.0006	0.0022
Female	433	59.64	0.0017	0.000	0.0012	0.0029
Female-Male			0.0006	0.074	-0.0001	0.0021

Supplementary Table 17 Mediation effects of APOE-ε4 and sex in the replication sample (n = 726). Mediation effects for the *Aβ* → Tau_{EC} → Tau_{Meta} pathway are shown across groups defined by APOE-ε4 allele count (0,1,2) (Part A) and sex (Part B). Weighted average mediation effects (WAME) were computed for each group by aggregating fully stratified estimates using empirical sample proportions. Pairwise contrasts (e.g., 1–0, 2–0, 2–1) indicate between-group differences; for instance, APOE-ε4 = 1–0 in Part A represents the WAME for APOE-ε4 heterozygotes minus that for non-carriers. One-sided p-values and one-sided 95% confidence intervals were computed using 1,000 multiplier bootstrap replications.

	$GVIF^{1/(2Df)}$ discovery	$GVIF^{1/(2Df)}$ replication
**Aβ as outcome**		
Age	1.04	1.01
Sex	1.02	1.01
TREM2	1.00	
APOE-ε4	1.01	1.00
**EC tau as outcome**		
Aβ	1.73	1.89
Age	1.06	1.08
Sex	1.50	1.12
TREM2	1.69	
APOE-ε4	1.75	1.28
Aβ ×APOE-ε4	1.96	1.45
Aβ × Sex	1.75	1.71
Sex ×TREM2	1.72	
**Meta Temp tau as outcome**		
Aβ	1.77	1.89
EC tau	1.88	1.96
Age	1.06	1.08
Sex	6.19	6.11
TREM2	6.43	
APOE-ε4	5.63	4.80
Aβ≥60 × EC tau	1.78	1.89
EC tau × APOE-ε4	5.76	4.88
EC tau × TREM2	6.74	
TREM2 × APOE-ε4	1.39	
Aβ × TREM2	1.91	
EC tau × Sex	6.39	6.25

Supplementary Table 18 Scaled generalized variance inflation factors (GVIF) for structural equation models in the discovery and replication samples. $GVIF^{1/(2Df)}$ values are reported for each covariate in the structural equation models predicting Aβ, entorhinal cortex (EC) tau, and meta-temporal tau. This metric is used to assess multicollinearity among predictors. Columns represent results from the discovery (n = 626) and replication (n = 726) cohorts, respectively. Missing entries reflect variables not included in the replication models.

Supplementary Methods

Model Building

To understand the heterogeneous influence of *APOE*-ε4, Sex, and *TREM2* on the downstream AD cascade, we adopted structural equation models (SEM) with interaction terms to estimate a variety of causal effects. While interaction terms here are helpful to capture the heterogeneity between subpopulations with different genetic profiles, given potentially limited statistical powers to model all possible interactions among variables presented in **Figure 1**, we adopted forward selection with the Akaike Information Criterion (AIC) to choose interaction terms for the SEMs. This approach sequentially evaluates and incorporates up-to-two-way interactions, ensuring that only those with substantial statistical contributions are included. To allow for an accurate estimation and interpretation of the causal effects when two continuous variables interact (i.e. Aβ and EC tau), we discretise Aβ into four bins (< 10, 10–40, 40–60, > 60) when including it as an interactive term with EC tau. Continuous Aβ is still included as a main effect variable when interactions are retained following model selection. This model selection procedure resulted in the following structural equation models:

$$\mathrm{A\beta} = \mu_o + \alpha_0\mathrm{Age} + \beta_0\mathrm{Sex} + \gamma_0 \mathit{TREM2} + \delta_{01}\, \mathit{APOE}\text{-}\varepsilon4_1 + \delta_{02}\, \mathit{APOE}\text{-}\varepsilon4_2 + \epsilon_0,$$

$$\mathrm{Tau_{EC}} = \mu_1 + \theta_1\mathrm{A\beta} + \alpha_1\mathrm{Age} + \beta_1\mathrm{Sex} + \gamma_1\mathit{TREM2} + \delta_{11}\, \mathit{APOE}\text{-}\varepsilon4_1 + \delta_{12}\, \mathit{APOE}\text{-}\varepsilon4_2 + \lambda_{11}\, \mathrm{A\beta} \times \mathit{APOE}\text{-}\varepsilon4_1 + \lambda_{12}\, \mathrm{A\beta} \times \mathit{APOE}\text{-}\varepsilon4_2 + \zeta_1\, \mathrm{A\beta} \times \mathrm{Sex} + \phi_1\mathrm{Sex} \times \mathit{TREM2} + \epsilon_1,$$

$$\mathrm{Tau_{Meta}} = \mu_2 + \theta_2\mathrm{A\beta} + \kappa_2\, \mathrm{Tau_{EC}} + \alpha_2\mathrm{Age} + \beta_2\mathrm{Sex} + \gamma_2\mathit{TREM2} + \delta_{21}\, \mathit{APOE}\text{-}\varepsilon4_1 + \delta_{22}\, \mathit{APOE}\text{-}\varepsilon4_2 + \nu_2 1_{\{\mathrm{A\beta}\geq 60\}} \times \mathrm{Tau_{EC}} + \tau_2\mathit{TREM2} \times \mathrm{Tau_{EC}} + \pi_{21}\, \mathit{APOE}\text{-}\varepsilon4_1 \times \mathrm{Tau_{EC}} + \pi_{22}\, \mathit{APOE}\text{-}\varepsilon4_2 \times \mathrm{Tau_{EC}} + \omega_{21}\mathit{TREM2} \times \mathit{APOE}\text{-}\varepsilon4_1 + \omega_{22}\mathit{TREM2} \times \mathit{APOE}\text{-}\varepsilon4_2 + \psi_2\mathrm{A\beta} \times \mathit{TREM2} + \eta_2\mathrm{Sex} \times \mathrm{Tau_{EC.}} + \epsilon_2.$$

Mediation Effect Analysis

These structural equation models facilitates the quantification of the mediation effect of the Aβ → $\mathrm{Tau_{EC}}$ → $\mathrm{Tau_{Meta}}$ pathway, which can be expressed as:

$$\mu(A\beta \mid \mathit{TREM2}, \mathit{APOE}\text{-}\varepsilon4, \mathrm{Sex}, 1_{\{A\beta\geq 60\}})$$
$$= \mathrm{E}(\mathrm{Tau_{Meta}} \mid \mathit{TREM2}, \mathit{APOE}\text{-}\varepsilon4, \mathrm{Sex}, 1_{\{A\beta\geq 60\}}, A\beta = c, \mathrm{Tau_{EC}}(A\beta = a + 1)) - \mathrm{E}(\mathrm{Tau_{Meta}} \mid \mathit{TREM2}, \mathit{APOE}\text{-}\varepsilon4, \mathrm{Sex}, 1_{\{A\beta\geq 60\}}, A\beta = c, \mathrm{Tau_{EC}}(A\beta = a))$$
$$= (\kappa_2 + \nu_2 1_{\{A\beta\geq 60\}} + \tau_2\mathit{TREM2} + \pi_{21}\, \mathit{APOE}\text{-}\varepsilon4_1 + \pi_{22}\, \mathit{APOE}\text{-}\varepsilon4_2 + \eta_2\mathrm{Sex}) \times (\theta_1 + \lambda_{11}\, \mathit{APOE}\text{-}\varepsilon4_1 + \lambda_{12}\, \mathit{APOE}\text{-}\varepsilon4_2 + \zeta_1\mathrm{Sex})$$

Supplementary Table 19 and 20 display the effect sizes and corresponding p-values for the mediation effect within various subgroups defined by levels of Sex (male/female), *TREM2* (0/1), *APOE*-ε4 (0/1/2), and $1_{\{A\beta\geq 60\}}$, all of which are mutually exclusive with sample sizes and proportions provided.

To elucidate the mediation effect with respect to individual factors, we calculated the weighted

mean of the mediation effect for varying levels of *APOE*-ε4, *TREM2*, and Sex, as presented in **Supplementary Table 16 and 17**. Additionally, pairwise differences within each factor are reported.

Sex	*TREM2*	*APOE*-ε4	1{*Aβ*≥60}	Group Size	Group Prop (100%)	Effect Size	p-value
0	0	0	0	130	20.7668	0.0009	0.002
1	0	0	0	141	22.5240	0.0013	0.000
0	1	0	0	6	0.9585	0.0013	0.004
1	1	0	0	10	1.5974	0.0020	0.000
0	0	1	0	51	8.1470	0.0010	0.004
1	0	1	0	69	11.0224	0.0014	0.000
0	1	1	0	3	0.4792	0.0015	0.002
1	1	1	0	4	0.6390	0.0021	0.000
0	0	2	0	7	1.1182	0.0037	0.003
1	0	2	0	11	1.7572	0.0043	0.000
0	1	2	0	0	0.0000	0.0048	0.004
1	1	2	0	2	0.3195	0.0056	0.000
0	0	0	1	33	5.2716	0.0009	0.003
1	0	0	1	31	4.9521	0.0014	0.000
0	1	0	1	0	0.0000	0.0013	0.004
1	1	0	1	2	0.3195	0.0020	0.000
0	0	1	1	49	7.8275	0.0010	0.003
1	0	1	1	51	8.1470	0.0015	0.000
0	1	1	1	5	0.7987	0.0015	0.002
1	1	1	1	7	1.1182	0.0022	0.000
0	0	2	1	6	0.9585	0.0038	0.003
1	0	2	1	7	1.1182	0.0044	0.000
0	1	2	1	0	0.0000	0.0049	0.003
1	1	2	1	1	0.1597	0.0057	0.000

Supplementary Table 19 Mediation effects across all combinations of sex, TREM2, APOE-ε4, and 1{Aβ ≥ 60} in the discovery sample (n = 626). Mediation effect sizes and corresponding p-values for the *Aβ* → Tau_{EC} → Tau_{Meta} pathway are provided for each unique group defined by sex (0 = male, 1 = female), TREM2 carrier status (0 = non-carrier, 1 = carrier), APOE-ε4 allele count (0, 1, or 2), and an indicator variable for Aβ ≥ 60. Each row represents a unique group, with group size and proportion in the full cohort provided. The two-sided p-values were computed using 1,000 multiplier bootstrap replications.

Sex	*APOE*-ε4	1{*Aβ*≥60}	Group Size	Group Prop (100%)	Effect Size	one-sided p-value
0	0	0	193	26.58	0.0009	0.003
1	0	0	294	40.50	0.0016	0.002
0	1	0	65	8.95	0.0010	0.001
1	1	0	94	12.95	0.0017	0.000
0	2	0	9	1.24	0.0034	0.082
1	2	0	8	1.10	0.0044	0.047
0	0	1	16	2.20	0.0011	0.002
1	0	1	20	2.75	0.0019	0.001
0	1	1	7	0.96	0.0013	0.001
1	1	1	16	2.20	0.0021	0.000
0	2	1	3	0.41	0.0040	0.070
1	2	1	1	0.14	0.0051	0.039

Supplementary Table 20 Mediation effects across all combinations of sex, APOE-ε4, and 1{Aβ ≥ 60} in the replication sample (n = 726). Mediation effect sizes and corresponding p-values for the *Aβ* → Tau_{EC} → Tau_{Meta} pathway are provided for each unique group defined by sex (0 = male, 1 = female), APOE-ε4 allele count (0, 1, or 2), and an indicator variable for Aβ ≥ 60. Each row represents a unique group, with group size and proportion in the full cohort provided. The one-sided p-values were computed using 1,000 multiplier bootstrap replications.
