# Supplementary material for: Variable and interactive effects of Sex, APOE ε4 and TREM2 on the deposition of tau in entorhinal and neocortical regions
Source: Nat Commun. 2025 Jul 1;16:5812. doi: 10.1038/s41467-025-60370-8 (PMC12214702; doi:10.1038/s41467-025-60370-8)
Supplement: Supplementary file 2 — Reporting Summary [file 41467_2025_60370_MOESM2_ESM.pdf]

nature portfolio

Corresponding author(s): Joseph Giorgio

Last updated by author(s): 2025/05/05

Reporting Summary

Nature Portfolio wishes to improve the reproducibility of the work that we publish. This form provides structure for consistency and transparency in reporting. For further information on Nature Portfolio policies, see our Editorial Policies and the Editorial Policy Checklist.

Statistics

For all statistical analyses, confirm that the following items are present in the figure legend, table legend, main text, or Methods section.

n/a	Confirmed	
☐	☒	The exact sample size (n) for each experimental group/condition, given as a discrete number and unit of measurement
☐	☒	A statement on whether measurements were taken from distinct samples or whether the same sample was measured repeatedly
☐	☒	The statistical test(s) used AND whether they are one- or two-sided<br>*Only common tests should be described solely by name; describe more complex techniques in the Methods section.*
☐	☒	A description of all covariates tested
☐	☒	A description of any assumptions or corrections, such as tests of normality and adjustment for multiple comparisons
☐	☒	A full description of the statistical parameters including central tendency (e.g. means) or other basic estimates (e.g. regression coefficient) AND variation (e.g. standard deviation) or associated estimates of uncertainty (e.g. confidence intervals)
☐	☒	For null hypothesis testing, the test statistic (e.g. F, t, r) with confidence intervals, effect sizes, degrees of freedom and P value noted<br>*Give P values as exact values whenever suitable.*
☒	☐	For Bayesian analysis, information on the choice of priors and Markov chain Monte Carlo settings
☒	☐	For hierarchical and complex designs, identification of the appropriate level for tests and full reporting of outcomes
☒	☐	Estimates of effect sizes (e.g. Cohen's d, Pearson's r), indicating how they were calculated

Our web collection on statistics for biologists contains articles on many of the points above.

Software and code

Policy information about availability of computer code

Data collection	Data were collected using the software and protocols as listed in the online information from each of the large publicly available datasets.
Data analysis	Image segmentation was performed using Freesurfer (V5.3), additional image processing pipelines that were written in MATLAB R2023a.<br>Structural Equation Models were run in R version 4.1.1 environment using standard packages.<br>SNP data were extracted using BCFTOOLs.

For manuscripts utilizing custom algorithms or software that are central to the research but not yet described in published literature, software must be made available to editors and reviewers. We strongly encourage code deposition in a community repository (e.g. GitHub). See the Nature Portfolio guidelines for submitting code & software for further information.

Data

Policy information about availability of data

All manuscripts must include a data availability statement. This statement should provide the following information, where applicable:

- Accession codes, unique identifiers, or web links for publicly available datasets
- A description of any restrictions on data availability
- For clinical datasets or third party data, please ensure that the statement adheres to our policy

ADNI, HABS-HD, and A4 studies data are available upon access approval through
the following websites. ADNI website: https://adni.loni.usc.edu/. HABS-HD website: https://apps.unthsc.edu/itr/. A4 website: https://atri.usc.edu/study/a4-study/.

Source data for the marginal effects shown in figures are provided with this paper.

Research involving human participants, their data, or biological material

Policy information about studies with human participants or human data. See also policy information about sex, gender (identity/presentation), and sexual orientation and race, ethnicity and racism.

Reporting on sex and gender	Sex: Female and Male are used referring to the biological attribute.
Reporting on race, ethnicity, or other socially relevant groupings	Self reported race was used as a proxy for ethnic differences due to either social or ancestral variation.
Population characteristics	Discovery sample (n=628, 79% cognitively normal, Age mean±std 74.6±6.8, Sex n(F%) 338(54%)). Validation sample sample (n=726, 76% cognitively normal, Age mean±std 65±8.1, Sex n(F%) 433(60%)). Additional characteristic listed in Table 1.
Recruitment	Participants were recruited based on multiple characteristic as defined by each of the respective cohorts. In pooling and validating using external cohorts we aimed to mitigate potential recruitment biases of an individual cohort.
Ethics oversight	This study was approved by the Institutional Review Boards of the University of California, Berkeley. For Alzheimer's Disease Neuroimaging Initiative (ADNI), Anti-Amyloid Treatment in Asymptomatic Alzheimer's Disease (A4), and Health and Aging Brain Study-Health Disparities (HABS-HD), ethical approval was obtained by the relevant investigators.

Note that full information on the approval of the study protocol must also be provided in the manuscript.

Field-specific reporting

Please select the one below that is the best fit for your research. If you are not sure, read the appropriate sections before making your selection.

[x] Life sciences [ ] Behavioural & social sciences [ ] Ecological, evolutionary & environmental sciences

For a reference copy of the document with all sections, see nature.com/documents/nr-reporting-summary-flat.pdf

Life sciences study design

All studies must disclose on these points even when the disclosure is negative.

Sample size	1,354. Sample was the maximum number of available data points who had requisite multi modal imaging and genetic information.
Data exclusions	2 participants were excluded from the analyses (ADNI participants as they were flagged as outliers based on Cook's distance.
Replication	We performed a replication analysis on an independent external cohort. Showing broad agreement in two independent samples. Due to incomplete sampling of risk TREM2 SNPs in HABS we were unable to run analysis to replicate this finding.
Randomization	Not relevant as this is only observational data.
Blinding	All authors were blind to the collection of data. Image analyses were performed independent of aims and analysis of this project. Analyses were performed by an independent author from those who performed the initial project development, data aggregation and manuscript preparation.

Reporting for specific materials, systems and methods

We require information from authors about some types of materials, experimental systems and methods used in many studies. Here, indicate whether each material, system or method listed is relevant to your study. If you are not sure if a list item applies to your research, read the appropriate section before selecting a response.

Materials & experimental systems

n/a	Involved in the study	
[x]	[ ]	Antibodies
[x]	[ ]	Eukaryotic cell lines
[x]	[ ]	Palaeontology and archaeology
[x]	[ ]	Animals and other organisms
[x]	[ ]	Clinical data
[x]	[ ]	Dual use research of concern
[x]	[ ]	Plants

Methods

n/a	Involved in the study	
[x]	[ ]	ChIP-seq
[x]	[ ]	Flow cytometry
[x]	[ ]	MRI-based neuroimaging

Plants

Seed stocks

Report on the source of all seed stocks or other plant material used. If applicable, state the seed stock centre and catalogue number. If plant specimens were collected from the field, describe the collection location, date and sampling procedures.

Novel plant genotypes

Describe the methods by which all novel plant genotypes were produced. This includes those generated by transgenic approaches, gene editing, chemical/radiation-based mutagenesis and hybridization. For transgenic lines, describe the transformation method, the number of independent lines analyzed and the generation upon which experiments were performed. For gene-edited lines, describe the editor used, the endogenous sequence targeted for editing, the targeting guide RNA sequence (if applicable) and how the editor was applied.

Authentication

Describe any authentication procedures for each seed stock used or novel genotype generated. Describe any experiments used to assess the effect of a mutation and, where applicable, how potential secondary effects (e.g. second site T-DNA insertions, mosiacism, off-target gene editing) were examined.
